# Adsorption of aliphatic polyhydroxy carboxylic acids on gibbsite: pH dependency and importance of adsorbate structure

**DOI:** 10.1186/s12302-017-0129-6

**Published:** 2018-01-12

**Authors:** Tatjana Schneckenburger, Jens Riefstahl, Klaus Fischer

**Affiliations:** 1 Present Address: Environment Conservation Consultant, Annabergstraße 54, 55131 Mainz, Germany; 2 Present Address: Biolandhof Apfelbacher, 53332 Bornheim-Brenig, Germany; 3 Faculty VI-Regional and Environmental Sciences, Department of Analytical and Ecological Chemistry, University of Trier, Behringstr. 21, 54296 Trier, Germany

**Keywords:** Gibbsite, Polyhydroxy carboxylic acids, Gluconic acid, Tartaric acid, Adsorption, Sorption edge, Sorption isotherm, Surface complexation, Nuclear waste repository, Artificial cement pore water

## Abstract

**Background:**

Aliphatic (poly)hydroxy carboxylic acids [(P)HCA] occur in natural, e.g. soils, and in technical (waste disposal sites, nuclear waste repositories) compartments . Their distribution, mobility and chemical reactivity, e.g. complex formation with metal ions and radionuclides, depend, among others, on their adsorption onto mineral surfaces. Aluminium hydroxides, e.g. gibbsite [α-Al(OH)_3_], are common constituents of related solid materials and mimic the molecular surface properties of clay minerals. Thus, the study was pursued to characterize the adsorption of glycolic, threonic, tartaric, gluconic, and glucaric acids onto gibbsite over a wide pH and (P)HCA concentration range. To consider specific conditions occurring in radioactive wastes, adsorption applying an artificial cement pore water (pH 13.3) as solution phase was investigated additionally.

**Results:**

The sorption of gluconic acid at pH 4, 7, 9, and 12 was best described by the “two-site” Langmuir isotherm, combining “high affinity” sorption sites (adsorption affinity constants $K_{\text{L1}}$ > 1 L mmol^−1^, adsorption capacities < 6.5 mmol kg^−1^) with “low affinity” sites ($K_{\text{L2}}$ < 0.1 L mmol^−1^, adsorption capacities ≥ 19 mmol kg^−1^). The total adsorption capacities at pH 9 and 12 were roughly tenfold of that at pH 4 and 7. The S-shaped pH sorption edge of gluconic acid was modelled applying a constant capacitance model, considering electrostatic interactions, hydrogen bonding, surface complex formation, and formation of solved polynuclear complexes between Al^3+^ ions and gluconic acid. A Pearson and Spearman rank correlation between (P)HCA molecular properties and adsorption parameters revealed the high importance of the size and the charge of the adsorbates.

**Conclusions:**

The adsorption behaviour of (P)HCAs is best described by a combination of adsorption properties of carboxylic acids at acidic pH and of polyols at alkaline pH. Depending on the molecular properties of the adsorbates and on pH, electrostatic interactions, hydrogen bonding, and ternary surface complexation contribute in varying degrees to the adsorption process. Linear distribution coefficients *K*_d_ between 8.7 and 60.5 L kg^−1^ (1 mmol L^−1^ initial PHCA concentration) indicate a considerable mineral surface affinity at very high pH, thus lowering the PHCA fraction available for the complexation of metal ions including radionuclides in solution and their subsequent mobilization.

## Background

Sorption of aliphatic polyhydroxy carboxylic acids (PHCA) onto mineral surfaces influences their fate in natural and technical compartments. Depending on their coordination and sorption properties, they may also modify the distribution and mobility of metal ions including radionuclides in these compartments.

(P)HCA comprise a group of low molecular weight compounds that exhibit one or more carboxyl and at least two hydroxyl functions. The PHCA representatives tartaric and ketogluconic acids were detected in root and microbial exudates, contributing to nutrient uptake (phosphate, trace elements) from minerals and soils [1, 2]. The high alkaline pH in radioactive wastes solidified by cement addition initiates the hydrolysis of cellulosic materials releasing sugar and other polyhydroxy carboxylic acids with α-isosaccharinic acid as the major compound [3–6]. High microbial activities in natural environments, e.g. soils, result in fast turnover rates of these compounds and in subsequent low concentrations (lower µmol L^−1^ range typically). However, higher concentrations might occur in cementitious repositories, where strongly alkaline conditions minimize activities of microbes.

Due to the availability of at least three electron pair donating functional groups, (P)HCA act as polydentate ligands able to chelate metal ions in aqueous solutions. So far, as investigated, (P)HCA complex stability, e.g. of metal gluconates, is low or moderate at acidic or neutral pH, but increases by several orders of magnitude at strongly alkaline pH due to the deprotonation of hydroxyl groups and their subsequent participation in chelate formation [7–10]. Thus, the occurrence and potential interactions of natural chelating agents, i.e. (P)HCA, with radionuclides is of crucial importance for safety assessment of cementitious geological disposal facilities [6, 11, 12].

Gibbsite [α-Al(OH)_3_] is the thermodynamically most stable polymorph of aluminium hydroxide under environmental conditions [13]. It represents the octahedral layer of many clay minerals and the Al containing phase in cementitious materials commonly used in low-level radioactive waste disposal. From the two kinds (singly and doubly coordinated by Al) of surface aluminol groups (≡Al–OH), only the singly coordinated type, which accounts for roughly 5% of all surface functions, is expected to take part in surface acid–base reactions and in electrostatic interactions in the pH range between 4 and 10 [14, 15]. Furthermore, their Lewis acidity and reactivity towards ligand exchange is high, because edge-located Al ions are coordinatively unsaturated.

When investigating the sorption of organic acids onto Al minerals, one main topic is the pH influence on the distribution equilibrium. As for the adsorption of aliphatic and aromatic polycarboxylic acids, it was repeatedly found that the adsorbed amounts reached a maximum within the pH range of 4.0–5.5 and strongly decreased with increasing pH up to the isoelectric point (pH_IEP_) of the minerals [16–20]. This pH dependency of the adsorption maximum is usually explained by an optimal balance between the degree of the acid dissociation which increases with rising pH, depending on the respective pK_a_ values, and the number of positive charges on the mineral surface which concomitantly decreases. The pH influence on the sorption of an aromatic polyhydroxy compound (1,2,3-benzenetriol) greatly differed from that of the aromatic polycarboxylates. For the polyol, the maximum of the adsorption density showed a broad plateau shape near pH 7 and sorption remained significant even at pH_IEP_ in the alkaline pH range [20]. The sorption of the monosaccharides glucose and fructose on alumina was highest at pH_IEP_ and remained high at pH 12.3 [21]. Sugar alcohols, the reduced forms of the corresponding monosaccharides, adsorbed onto gibbsite even at pH 14 [22]. The adsorbed amounts increased with molecular size, the number of hydroxyl groups, and the fulfilment of specific steric constraints.

To develop a mechanistic understanding of surface reactions, e.g. surface complex formation, the pH-dependent ligand species distribution and the complex formation in solution need to be considered. Al^3+^ ions are coordinated by (P)HCA at pH < 10 via the carboxyl and the α-hydroxyl group, preferentially in a bidentate manner, accompanied by a stepwise deprotonation of further hydroxyl groups with increasing pH. At pH > 10, the coordinating carboxyl group is progressively replaced by a second, vicinal hydroxyl group sustaining bidentate chelation. If the required number of hydroxyl groups is available in suited steric positions as in gluconic acid, tridentate coordination is realized [9, 23].

The discussed types of surface binding are outer-sphere complexation (≡Al–OH_2_^+^L^−^) at high positive surface charge together with polar interactions, i.e. H bonding, and inner-sphere complexation, relevant within a wide pH range. A further hypothesized bonding mechanism is ternary surface complex formation with complexes already formed in solution. The relative importance of these mechanisms might vary not only with pH, but also with adsorbate properties, stoichiometric ratio of adsorbate molecules to surface sites, ionic strength, and dissolved Al ion concentrations.

To the best of our knowledge, sorption of (P)HCA on α-Al(OH)_3_ was only investigated by Coyne et al. [23], who did not consider the aspects of the sorption mechanism, and by Essington and Anderson [15], who focused on competitive adsorption of 2-ketogluconic acid and inorganic ligands.

To elucidate (P)HCA sorption on gibbsite, the (P)HCA phase distribution in gibbsite suspensions was determined and fitted by Langmuir and composite Freundlich–Langmuir isotherms in this study. The sorption kinetics and edges were measured for gluconic acid. Surface and adsorbate species at respective pH were assessed and evidence for the postulated mechanisms was gained by surface complexation modelling. The importance of molecular adsorbate properties for surface binding at pH 13.3 was examined. Compared with the adsorption properties of structurally related compounds, i.e. polyols and polycarboxylates, mutual and distinct reaction characteristics of (P)HCA have been outlined.

## Methods

### Materials

Four PHCA and glycolic acid (GLY, free acid, received from Merck, Darmstadt, Germany) were used for sorption experiments. The PHCA l-tartaric acid (TAR, sodium salt) and d-gluconic acid (GLU, sodium salt) were also purchased from Merck. d-glucaric acid (GLA, synonym: saccharic acid, potassium salt) came from Sigma Aldrich (Seelze, Germany) and d-threonic acid (THR, hemicalcium salt monohydrate) was supplied by Fluka (Taufkirchen, Germany). All the organic acids as well as the other chemicals were of analytical grade. Properties and Fischer projections of the compounds are given in Table 1.

**Table 1:** Properties of the (poly)hydroxy carboxylic acids

Compound name (acid)	Acronym	CAS-no.	Sum formula	Molar mass [g mol^−1^]	No. of OH	No. of COOH	pK_s1_	pK_s2_
Glycolic	GLY	76-14-1	C_2_H_4_O_3_	76.04	1	1	3.83	n.a.
d-Threonic	THR	70753-61-6	C_4_H_8_O_5_	136.10	3	1	3.86^a^	n.a.
l-Tartaric	TAR	87-69-4	C_4_H_6_O_6_	150.09	2	2	2.98	4.34
d-Gluconic	GLU	527-07-1	C_6_H_12_O_7_	196.16	5	1	3.86	n.a.
d-Glucaric	GLA	576-42-1	C_6_H_10_O_8_	210.14	4	2	3.17	3.97
				
Glycolic acid	d-Threonic acid		l-Tartaric acid		d-Gluconic acid		d-Glucaric acid	

n.a., not applicable

^a^Value of gluconic acid

A synthetic gibbsite MARTINAL^®^ OL-111/LE (Martinswerk GmbH, Bergheim, Germany) with a purity of 99.4% (data certified by supplier) was used without further pretreatment. The purity of the material was confirmed by X-ray diffraction (D500, Siemens AG, Munich, Germany) and scanning electron microscopy (LEO 435 Lv, Leo Elektronenmikroskopie GmbH, Oberkochen, Germany). The amount of surface hydroxyl groups and their acid–base properties were characterized by batch-back titration [24], using a PC-controlled titration system (751 GPD Titrino with software Vesuv 3.0, Metrohm AG, Herisau, Switzerland). For this purpose, suspensions of 1.0 g of gibbsite in 50 mL of NaClO_4_ solution were prepared, establishing three parallel series with 0.1, 0.01, and 0.001 M NaClO_4_ concentrations, respectively. Then diluted nitric acid or sodium hydroxide solutions were added in such a manner that every incremental pH value (0.1–0.2 pH unit increments) between 3.0 and 12.0 was adjusted using an individual sample for every specific pH value. The consumed acid or base amounts were registered. The sample vessels were end-over-end shaken overnight at room temperature. Afterwards, the phases were separated by centrifugation (5000*g*) and membrane filtration (0.2 µm). After pH determination, 25 mL of each supernatant was collected and slowly back titrated until the initial pH value was reached. Between any titre dosage, a hold time of 3 min was maintained. The amount of active surface hydroxyl groups was deduced from the differences between the titre amounts added during the first and the second titration step. The remaining amount of supernatant was used for the analytical determination of solved Al ions (AAS 3030, Perkin-Elmer, Überlingen, Germany). Based on these data, the amounts of gibbsite particles in the titrated solutions were calculated. Due to the low concentration of solved Al^3+^-ions in the pH range from 5.0 to 9.3, no correction of the titration data with respect to potential consumption of titre reagents by gibbsite solubilization was made.

### Sorption experiments

Sorption of the (P)HCA was determined in batch experiments at room temperature with equilibration times of 24 h. Batches contained 2 g of gibbsite suspended in 10 mL of background electrolyte, filled in centrifuge tubes made of Teflon or polyethylene. Experiments with GLU at varying pH values were conducted in 5 mmol L^−1^ NaClO_4_. An artificial cement pore water (ACPW) was used instead of NaClO_4_ in experiments conducted to examine the potential correlations between the structural features of (P)HCA and their sorption behaviour. The ACPW should mimic the pore water composition characteristically for some types and process stages of nuclear waste solidification. The ACPW contained the following ion concentrations: 114 mmol L^−1^ Na^+^, 180 mmol L^−1^ K^+^ and 2 mmol L^−1^ Ca^2+^, resulting in a total ionic strength of about 300 mmol L^−1^ and a pH of 13.3 [25]. All experiments using the ACPW medium were conducted in a glove box under a nitrogen atmosphere. Sorption isotherms were recorded for all (P)HCA at initial adsorbate concentrations between 0.01 and 250 mmol L^−1^, respectively, subdivided into 10–12 concentration levels. Isotherms at pH 4, 7, 9, and 12, respectively, were recorded for GLU dissolved in NaClO_4_ solutions. The background electrolyte NaClO_4_ was added to provide a moderate and constant ion concentration in solution even at very low GLU concentrations and to avoid extreme changes of the composition and extension of the electrical double layer at the gibbsite surface. Phase separation was done by centrifugation (5000*g*) and filtration through 0.2 µm cellulose acetate filters.

The sorption kinetics of GLU were investigated in 11 time steps from 0.5 to 96 h. The solid-to-solution ratio was 1 g:5 mL and an initial GLU concentration of 1 mmol L^−1^ was adjusted in the 5 mmol L^−1^ NaClO_4_ electrolyte. The resulting pH was 9.0, determined after 96 h. No buffer was added for pH stabilization.

The pH dependency of GLU sorption (sorption edge) was further determined from pH 3.4 to pH 12.2 at an initial GLU concentration of 1 mmol L^−1^. Gibbsite solubility in the suspensions was controlled by AAS.

The equilibrium concentrations of the (P)HCA in the supernatants were determined by ion exclusion chromatography. A Dionex (Sunnyvale, CA) DX-500 system was used equipped with an isocratic high pressure pump IP 20, a Dionex IonPac HPICE-AS 6 separation column, and a Shimadzu (Duisburg, Germany) SPD 10 AVP UV/VIS detector, wavelength set to 210 nm. 0.55 mmol L^−1^ H_2_SO_4_ served as eluent. Due to the fast lactonization of GLU under that conditions, the GLU signal corresponded mainly to the lactone form of the analyte. Equal lactonization degrees of the GLU calibration standard and of GLU containing samples were implied for identical analytical treatments. The sorbed amounts of (P)HCA were calculated from the difference between initial and equilibrium concentrations in solution.

Gibbsite surfaces were further analysed before and after surface coating with GLU at pH 4, 8, 10, and 12, respectively, by DRIFT (diffuse reflectance infrared Fourier transform) spectroscopy. Therefore, the materials were prepared in batches containing GLU in an initial concentration of 50 mmol L^−1^ together with the background electrolyte 100 mmol L^−1^ NaClO_4_. Coated gibbsite was freeze dried, ground, mixed with solid KBr (approximately, 300 mg KBr per 10 mg of sample), and measured under a continuous nitrogen stream at a wavelength resolution of 4 cm^−1^ with 40 scans on a Bruker IFS-66 spectrometer (Bruker Optics, Ettlingen, Germany) with a Harrick reflectance device, MCT detector, and KBr as reference material.

### Sorption isotherms and data analysis

Sorption isotherms were mostly S shaped and thus described by a two-site Langmuir equation (Eq. 1) [26, 27]. This isotherm combines two Langmuir terms and assumes two types of sorption sites,

1 $$c_{\text{s}} \left( {c_{\text{eq}} } \right) = \frac{{K_{{{\text{L}}1}} \cdot c_{{{\text{sat}}1}} \cdot c_{\text{eq}} }}{{1 + K_{{{\text{L}}1}} \cdot c_{\text{eq}} }} + \frac{{K_{{{\text{L}}2}} \cdot c_{{{\text{sat}}2}} \cdot c_{\text{eq}} }}{{1 + K_{{{\text{L}}2}} \cdot c_{\text{eq}} }},$$

where *C*_s_ is the sorbed concentration [mol kg^−1^], *C*_eq_ [mol L^−1^] the equilibrium concentration in the liquid phase, *K*_L_ [L mol^−1^] the sorption affinity constant, and *C*_sat_ [mol kg^−1^] the sorption capacity of the first (index 1) and the second (index 2) type of sorption sites.

Some sorption reactions were additionally fitted with the Freundlich isotherm which is defined as:

2 $$c_{{s({\text{Ceq}})}} = \, K_{\text{F}} \cdot c_{\text{eq}}^{n} ,$$

where *C*_s_ is the sorbed concentration [mol kg^−1^], *C*_eq_ [mol L^−1^] the equilibrium concentration in the liquid phase, *K*_F_ [mol^1−*n*^ L^*n*^ kg^−1^] the Freundlich coefficient, and *n* [−] the Freundlich exponent.

In addition, we applied a composite Langmuir–Freundlich isotherm which combines the parameter of the one-site Langmuir and of the Freundlich isotherm [27] (Eq. 3),

3 $$c_{\text{s}} \left( {c_{\text{eq}} } \right) = \frac{{K_{\text{L}} \cdot c_{\text{sat}} \cdot c_{\text{eq}} }}{{1 + K_{\text{L}} \cdot c_{\text{eq}} }} + K_{\text{F}} \cdot c_{\text{eq}}^{n} .$$

Furthermore, the following adsorption isotherms and models had been tested, but did not yield satisfying results: the one-site Langmuir isotherm, the BET isotherm, and the dual mode model [28].

Sorption kinetics were described by first- and second-order kinetic models. A two-stage kinetic model (TSKM, Eq. 4) was applied,

4 $$c_{\text{s}} \left( t \right) = c_{\text{eq}} \cdot \frac{t}{{\frac{1}{{k_{1} }} + t}} + 2 \cdot k_{2} \cdot t^{0.5} ,$$

where *C*_s_(*t*) is the adsorbed concentration [mol kg^−1^] at the time *t* [h], *C*_eq_ [mol kg^−1^] the equilibrium concentration in the liquid phase (sorption process), *k*_1_ [s^−1^] the fast sorption rate constant, and *k*_2_ [mol kg^−1^ s^−0.5^] the slow or diffusion rate [29].

Fitting was done with Origin 7.0 (OriginLab Corporation, Northhampton, MA, USA) by non-linear least squares fitting using the Levenberg–Marquardt algorithm. During the fitting procedure, the relative error *R*_e_ was weighted by a factor of 1/*c*_s_, selecting the option ‘statistical weighting’ in Origin. Due to this weighting technique, errors relative to the data value were regarded instead of absolute errors and errors in the low concentration range become large.

Furthermore, the coefficient of determination (*r*^2^) and the goodness of fit at a given degree of freedom (*X*^2^/DOF) were calculated for every model application. The lower the *X*^2^/DOF value, the higher is the fitting quality.

### Modelling

Surface speciation of gibbsite was conducted with FITEQL (version 4.0) based on the batch-back titration data using the constant capacitance and the diffuse double-layer model [30]. The adsorption edges were modelled by a constant capacitance model using the following input data: gibbsite concentration: 20 g L^−1^, [≡SOH]: 120 mmol kg^−1^ (at a gibbsite concentration of 20 g L^−1^ corresponding to 2.4 mmol L^−1^), surface area: 11 m^2^ g^−1^, *I*_0_: 0.1 mol L^−1^, and volume of aqueous phase: 0.05 L.

## Results

### Gibbsite characteristics

Table 2 lists the properties of the gibbsite specimen used in this study and, for comparison, gibbsite characteristics stemming from other studies [31]. The specific surface area of our material with 11 m^2^ g^−1^ was in the lower range of the reference values, but the number of surface sites (6–7 per nm^2^) and surface speciation data, i.e. *K*_intr_ and pH_IEP_, coincided with literature data very well. Applying the constant capacitance model, it was established that most of the aluminol groups were uncharged in the pH range from 3 to 13, which indicates a high proportion of doubly coordinated surface functions.

**Table 2:** Surface and mineralogical properties of gibbsite

Property	Measured and modelled data	Ref. [31]
Purity^a^	99.4%	
Density^a^	2.4 g cm^−3^	
Particle size^a^ (laser diffraction)	D_10_ 0.3–0.5 µm	
	D_50_ 0.7–1.2 µm	
	D_90_ 1.2–2.7 µm	
Surface area^a^	10–12 m^2^ g^−1^	32 ± 25 m^2^ g^−1b^
Surface sites [≡SOH] (titration data)	120 mmol kg^−1^, 2.4 mmol L^−1^ (in batch)	8.2 ± 0.3 sites nm^−2c^
	6.02–7.23 sites nm^−2^, mean: 6.57 sites nm^−2^	
Isoelectric point (pH_IEP_)	Diffuse double-layer model: pH: 8.28	9.1 ± 0.7^d^
	Constant capacitance model: pH: 8.04	
Surface protonation constants	Diffuse double-layer model: pK_intr_ (+): 6.60, pK_intr_(−): − 9.96	pK_intr_ (+): 7.17^e^
	Constant capacitance model: pK_intr_ (+): 5.66, pK_intr_(−): − 10.41	pK_intr_ (−): − 11.18^e^
pH (24 h, 5 mM NaClO_4_)	9.12 ± 0.01	

^a^Data provided by manufacturer

^b^Mean of 42 BET/N_2_ values, p. 60/61

^c^Mean of 6 values, p. 64

^d^Mean of 19 values, p. 64

^e^Best fits obtained from double-layer model (including 13 titration curves from several studies)

The solved Al(III)_tot_ concentration was ≤ 10^−5^ M in the pH range 5.0–9.3 after suspending gibbsite during 24 h in NaClO_4_ solutions (*I*_0_: 01. and 0.01 M) and reached 10^−3^ M at pH 11.5. This information is relevant for the interpretation of sorption results, because it can be related to the potential loss of sorbent material, to the formation of Al–(P)HCA complexes in solution and to the potential precipitation of sparingly soluble Al–(P)HCA compounds. The solubility increases at pH > 9.5, presumably caused by the formation of Al–hydroxo complexes, matched with reference data [32]. At low pH, the experimental gibbsite dissolution was much lower than expected (0.5 mM at pH 2.2), which may suggest that dissolution equilibrium was not reached within 24 h. The ionic strength of the background electrolyte did not affect gibbsite dissolution within the error margins. Loss of solid phase in the batch experiments was negligible at pH 7.8 with about 0.0004% of the total particle mass, but reached 1.2% at pH 12.

### Sorption of gluconic acid

#### Sorption kinetics

**Fig. 1:**
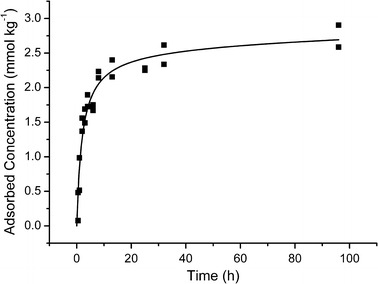
Kinetics of d-gluconic acid adsorption onto gibbsite at pH 9 in 5 mmol L^−1^ NaClO_4_ solution. Data points: experimental values, solid line: two-stage kinetic model (TSKM), model fit: *r*^2^: 0.931, *X*^2^/DOF: 0.041

The kinetics of GLU sorption onto gibbsite was determined to elucidate the timescale of the process and to obtain the batch shaking time necessary for establishing sorption equilibrium. Sorption kinetics helps to identify non-ideal sorption modes that are indicated by the occurrence of slow sorption processes. Sorption kinetics of GLU over 96 h at pH 9 neither clearly followed a first- nor a second-order sorption mechanism (Fig. 1, plots of *t* ~ ln *c* and *t*^−1^ ~ ln *c* were not linear), elucidating the involvement of more than one sorption mechanism. The two-stage kinetic model (Eq. 4) described the data well (*r*^2^: 0.931) and yielded an equilibrium concentration of the fast adsorbing GLU fraction of 2.47 ± 0.27 mmol kg^−1^ at a rate constant (*K*_1_) of 0.50 ± 0.11 h^−1^. The equilibrium was established after 30 h. A slight increase in sorption at longer times, represented by the slow sorption rate *k*_2_, was not significant (*k*_2_ 0.014 ± 0.017 mmol kg^−1^ h^−0.5^).

#### pH dependence of the sorption isotherms

**Fig. 2:**
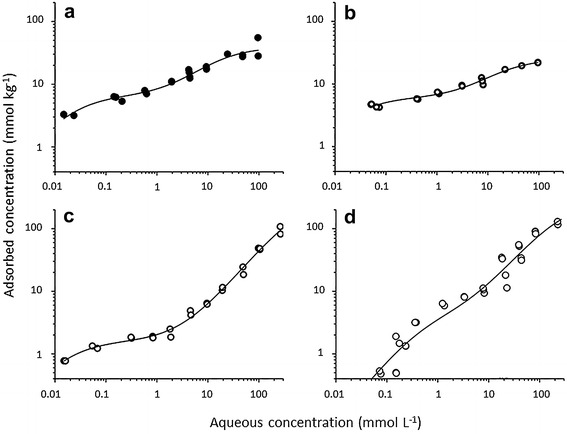
Sorption isotherms of d-gluconic acid at four pH values (**a** pH 4, **b** pH 7, **c** pH 9, **d** pH 12). Isotherms were recorded in 5 mM NaClO_4_ solution. Best fits were obtained by the two-site Langmuir isotherm. The data correspond to equilibrium concentrations

Isotherms of the sorption of GLU solved in 5 mM NaClO_4_ electrolyte solutions showed a weak S-shaped curvature at all pH values (Fig. 2). The isotherms were best described by the two-site Langmuir model (Eq. 1, Table 3, *r*^2^: 0.91–0.98). In addition, the data were fitted by the Freundlich isotherm almost equally well (*r*^2^: 0.91–0.97), since the S shape was weakly pronounced only (Table 4). Though there was no distinct correlation of any of the isotherm parameters (*K*_L_, *C*_sat_, *K*_F_) with pH, systematic changes in sorption behaviour of GLU with pH were observed.

**Table 3:** Two-site Langmuir isotherm fitting parameters for sorption of gluconic acid on gibbsite at various pH values

pH	*χ*^2^/DoF	*r* ^2^	*K*_L1_ ± *S*_D_ L mmol^−1^	*C*_sat1_ ± *S*_D_ mmol kg^−1^	*K*_L2_ ± *S*_D_ L mmol^−1^	*C*_sat2_ ± *S*_D_ mmol kg^−1^
4	0.674	0.912	51 ± 51	6.4 ± 1.5	0.069 ± 0.032	33 ± 5
7	0.072	0.979	48 ± 76	6.0 ± 1.8	0.053 ± 0.045	19 ± 5
9	0.362	0.981	63 ± 111	1.57 ± 0.57	0.0015 ± 0.0009	325 ± 149
12	2.40	0.920	3.70 ± 2.05	2.05 ± 1.91	0.0038 ± 0.0012	274 ± 57

*X*^2^/DoF, probability distribution normalized to the number of data points; *r*^2^, determination coefficient; *K*_L_, Langmuir affinity constant (1: “high”, 2: “low” affinity sites); *C*_sat_, saturation concentration; *S*_D_, standard deviation

**Table 4:** Freundlich isotherm fitting parameters for sorption of gluconic acid on gibbsite at various pH values

pH	*χ*^2^/DoF	*r* ^2^	*K*_F_ ± *S*_D_ [mmol^1 – *n*^ L^*n*^ kg^−1^]	*n* ± *S*_D_
4	0.61	0.912	9.33 ± 0.78	0.30 ± 0.03
7	0.11	0.965	7.48 ± 0.77	0.23 ± 0.03
9	0.67	0.962	1.20 ± 0.25	0.78 ± 0.04
12	2.56	0.908	2.91 ± 0.36	0.70 ± 0.03

*K*_F_, Freundlich constant; *n*, Freundlich exponent; *S*_D_, standard deviation; *χ*^2^/DoF, probability distribution normalized to the number of data points; *r*^2^, determination coefficient

The saturation concentration for the first type of sorption sites (“high affinity sites”, *C*_sat1_) was about three times greater at pH 4 and 7 than at pH 9 and 12, whereas the concentration of the second type of sorption sites (“low affinity sites”, *C*_sat2_) was roughly one order of magnitude higher at alkaline pH than at neutral or moderately acidic pH. The affinity constant related to the high affinity sites (*K*_L1_) was not significantly different between pH 4 and 9 (range 48–63 L mol^−1^), but was essentially lower at pH 12 (3.70 ± 2.05 L mol^−1^). *K*_L1_ was three or four orders of magnitude higher than *K*_L2_ at all pH values. At pH 9, the differences between both the affinity constants and between the saturation concentrations of the two sorption sites were greatest.

#### Surface complexation modelling

**Fig. 3:**
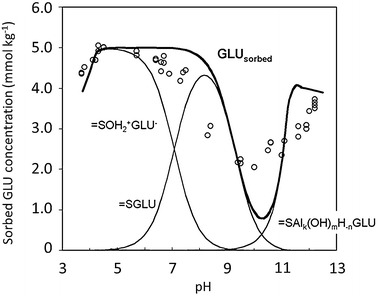
pH sorption edge of d-gluconic acid and sorption modelling. Initial d-gluconic acid concentration in the aqueous phase 1 mmol L^−1^, background electrolyte 0.1 M NaClO_4_, gibbsite suspension concentration 20 g L^−1^; circles: experimental data

The pH dependency of GLU sorption (sorption edge) was recorded from pH 3.4 to 12.2 at a constant initial GLU concentration of 1.0 mmol L^−1^ (Fig. 3) and modelled in FITEQL. The initial concentration corresponded to the lower concentration range of the isotherms presented above. The sorption edge was S shaped with a maximum around pH 4 and a minimum around pH 10. The decrease of the adsorbed amounts in the sequence pH 4 > pH 7 > pH 9 matched almost exactly the parallel decrease of the surface concentration of the “high affinity” sites (*C*_sat1_), noticed in Fig. 2.

For modelling, we considered dissociation of GLU, surface speciation of gibbsite, outer-sphere surface complexation by electrostatic forces, and inner-sphere complexation by ligand exchange. We included dissolution of gibbsite and the formation of soluble AlGLU complexes [9]. We took complexes with the general formula [Al_k_(H_−*n*_GLU)(OH)_*m*_]^(*n*+1+*m*−3*k*)−^ as potential adsorbates into account, allowing for polynuclear complex formation. There were no thermodynamic data available for the estimation of the relative abundance of those complexes. Furthermore, the conversion of GLU into of one of its lactonic forms under acidic pH had to be respected. Here, adsorption by H bonding and polar interactions seems to be very likely. Since the pH-dependent conversion degrees were not known, this aspect was not quantitatively treated. All model reactions are listed in Table 5.

**Table 5:** Speciation, dissolution, and complexation reactions and reaction constants involved in the sorption of gluconic acid to gibbsite within the pH range of 3.4–12.2

Reaction	Reaction constant lg K
Dissociation of gluconic acid	
HGLU $\Leftrightarrow$ H^+^ + GLU^−^	− 3.86
Surface speciation of gibbsite (constant capacitance model)	
≡SOH + H^+^ $\Leftrightarrow$ ≡SOH_2_^+^	5.66^a^
≡SOH $\Leftrightarrow$ ≡SO^−^ + H^+^	− 10.41^a^
Outer-sphere complexation	
≡SOH_2_^+^ + GLU^−^ $\Leftrightarrow$ ≡SOH_2_^+^ GLU^−^	13.93^a^
Inner-sphere complexation	
≡SOH + GLU^−^ $\Leftrightarrow$ ≡SGLU + OH^−^	6.92^a^
Dissolution of gibbsite	
Al(OH)_3_(s) + 3 H^+^ $\Leftrightarrow$ Al^3+^ 3 H_2_O	8.50^b^
Al^3+^ + OH^−^ $\Leftrightarrow$ AlOH^2+^	3.53^b^
Al^3+^ + 2 OH^−^ $\Leftrightarrow$ Al(OH)_2_^+^	− 0.80^b^
Al^3+^ + 3 OH^−^ $\Leftrightarrow$ Al(OH)_3_	− 6.50^b^
Al^3+^ + 4 OH^−^ $\Leftrightarrow$ Al(OH)_4_^−^	− 14.50^b^
Formation of Al–gluconate complexes	
Al^3+^ + GLU^−^ $\Leftrightarrow$ [AlGLU]^2+^	1.98^c^
Al^3+^ + GLU^−^ $\Leftrightarrow$ [Al_H − 1_GLU]^+^ + H^+^	− 0.89^c^
Al^3+^ + GLU^−^ $\Leftrightarrow$ [Al_H − 3_GLU]^−^ + 3 H^+^	− 10.18^c^
Formation of mixed Al–hydroxo-gluconate complexes (*k*: 1 or 2)	
*k* Al(OH)_4_^−^ + GLU^−^ $\Leftrightarrow$ [Al_*k*_(OH)_[3*k*+(*k* − 1) − *n*]_H_−*n*_GLU]^*k*−^ + OH^−^ + *n* H_2_O	9.80^d^
Sorption of the Al–hydroxo-gluconate complexes by inner-sphere complexation	
≡SOH + [Al_*k*_(OH)_[3*k*+(*k* − 1) − *n*]_H_−*n*_GLU]^*k*−^$\Leftrightarrow$ ≡S[Al_*k*_(OH)_[3*k*+(*k* − 1) − *n*]_H_−*n*_GLU]^(*k* − 1)−^ + OH^−^	7.09^d^

Further model parameters: capacity of the Helmholtz layer: 5 F m^−2^; solid-to-liquid ratio: 200 g L^−1^; specific surface area: 11 m^2^ g^−1^; initial gluconate concentration: 1 mmol L^−1^: ionic strength: 100 mmol L^−1^, density of surface sites: 5.5 sites nm^−2^

^a^Results of surface speciation modelling (FITEQL, constant capacitance model)

^b^[32]

^c^*I*_0_ 0.1 M KNO_3_, *T* 25 °C [9]

^d^Results of gluconate sorption edge modelling (FITEQL, constant capacitance model)

Outer-sphere complexation (electrostatic interactions) dominated in the acidic to neutral pH range. Match of data and model was high in this pH range. The adsorption decline at pH < 4 was likely provoked by the decreasing dissociation of GLU and by its transformation into the lactonic form.

Between pH 7 and 9, the model predicted inner-sphere complex formation. The model overestimated the data in this pH range which may relate to the fact that it was not equipped with a code allowing for the calculation of equilibrium concentrations of GLU surface species formed by polar interactions, e.g. hydrogen bonding. DRIFT spectra did not indicate inner-sphere surface complexation (Table 6). The model predicted the onset of ternary surface complex formation around pH 9, underestimating adsorption between pH 9.5 and 11. The cause of this underestimation remains speculative. Presumably, further AlGLU species were formed in solution and subsequently adsorbed which were not considered by the model. At pH 12 the contribution of ternary surface complex formation to sorption was significant. The formation of covalent bonds upon GLU sorption at high pH was supported by the DRIFT spectra. Particles that were covered by GLU at pH 12 showed a shift of the asymmetric valence vibration of the carboxylate group to lower wave numbers, indicating their participation in a coordinative bond. Since the carboxylate group is not involved in complex formation in solution at high pH, it is available for surface binding of Al–(P)HCA compounds.

**Table 6:** Wavenumbers *ν* [cm^−1^] of asymmetric and symmetric carboxylate stretching vibrations of GLU adsorbed on gibbsite, determined by DRIFT spectroscopy

pH	GLU on gibbsite		GLU on gibbsite—difference spectra	
	*ν*_asymmetric_ (COO^−^)	*ν*_symmetric_ (COO^−^)	*ν*_asymmetric_ (COO^−^)	*ν*_symmetric_ (COO^−^)
4	1644	1393	1643	1397
8	1650	1393	1652	1397
10	1650	1396	1646	1400
12	*1630*	1393	*1626*	–^a^

^a^Peak evaluation impossible

## Adsorption of (P)HCAs onto gibbsite suspended in artificial cement pore water

**Fig. 4:**
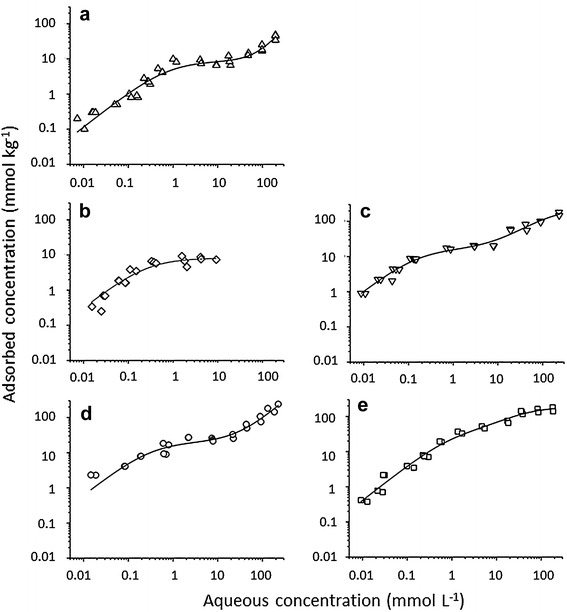
Sorption isotherms of glycolate (**a**), threonate (**b**), tartrate (**c**), gluconate (**d**), and glucarate (**e**) determined in the gibbsite/ACPW system at pH 13.3. Fitted isotherm functions (lines): glycolate and gluconate: composite Langmuir–Freundlich (parameters: Table 8), threonate: Langmuir (parameters: Table 7), tartrate and glucarate: two-site Langmuir. The data correspond to equilibrium concentrations. Isotherm parameters for tartrate: *X*^2^/DOF: 1.39, *r*^2^: 0.96, *K*_L1_: 6.6 ± 2.2 L mmol^−1^, *C*_sat1_: 16.1 ± 2.7 mmol kg^−1^, *K*_L2_: 0.007 ± 0.002 L mmol^−1^, *C*_sat2_: 228 ± 33 mmol kg^−1^. Isotherm parameters for glucarate: *X*^2^/DOF: 1.66, *r*^2^: 0.97, *K*_L1_: 1.0 ± 0.5 L mmol^−1^, *C*_sat1_: 36 ± 13 mmol kg^−1^, *K*_L2_: 0.03 ± 0.01 L mmol^−1^, *C*_sat2_: 154 ± 14 mmol kg^−1^

The specific characteristics of the sorption behaviour of the (P)HCA were deduced from equilibrium data of the (P)HCA distribution in the gibbsite/ACPW (artificial cement pore water) system at pH 13.3 ± 0.30. Due to the high ionic strength of the ACPW (I_0_ ≈ 300 mmol L^−1^), electrostatic interactions at the interface were strongly suppressed. Calculating THR isotherms, equilibrium concentrations ≥ 20 mmol L^−1^ were excluded because of the formation of precipitates at these concentrations. The isotherms were S shaped for all (P)HCA except THR. The S shape was more pronounced for the monocarboxylic acids than for the dicarboxylic acids (Fig. 4). In cases were the Langmuir isotherm was not suited to fit the data of the monocarboxylic acids, the composite Langmuir–Freundlich isotherm was applied. All acids showed a Langmuir-type sorption behaviour up to equilibrium concentrations of approximately 10–20 mmol L^−1^, suggesting similar adsorption mechanism. The values of *C*_sat_ and *K*_L_ of the two composite models (2-site Langmuir and Langmuir–Freundlich) may not be directly compared. Thus, the Langmuir model was used to describe the first isotherm branch up to equilibrium concentrations of ≤ 20 mmol L^−1^ (Table 7).

**Table 7:** Langmuir isotherm parameters of the (P)HCA adsorption onto gibbsite in the ACPW medium

(P)HCA	*χ*^2^/DoF	r^2^	*K*_L_ ± *S*_D_ [L mol^−1^]	*C*_sat_ ± *S*_D_ [mol kg^−1^]
GLY	0.34	0.894	1.30 ± 0.43	8.9 ± 1.3
THR	0.31	0.900	4.0 ± 1.60	8.1 ± 1.3
TAR	0.12	0.981	4.62 ± 1.15	21.2 ± 2.3
GLU	1.00	0.872	1.87 ± 0.59	25.9 ± 3.0
GLA	0.38	0.970	0.57 ± 0.14	67.7 ± 9.6

Reduced data set (*C*_eq_ ≤ 20 mmol L^−1^)

*K*_L_, Langmuir affinity constant; *C*_sat_, saturation concentration; *S*_D_ standard deviation; *χ*^2^/DoF probability distribution normalized to the number of data points; *r*^2^ determination coefficient

Applying this model, the *C*_sat_ levels of all (P)HCA were below the total number of surface sites (120 mmol kg^−1^) as for GLU at all pH conditions. The surface concentrations (*C*_sat_) increased with increase in the molecular size and the number of carboxylic acid groups of the adsorbates.

The *C*_sat_ values of both dicarboxylic acids (TAR and GLA) were roughly threefold that of the corresponding monocarboxylic acids (THR and GLU).

To attain a consistent data set including phase distribution at high ligand concentration and to generate additional sorption parameters useful for the following correlation analysis, linear distribution coefficients were calculated for initial (P)HCA concentrations of 1.0 and 100 mmol L^−1^, respectively. Furthermore, the composite Langmuir–Freundlich isotherm was applied (Table 8). For the Langmuir part of the isotherm, the fitting results were in as far consistent with the one-site Langmuir isotherm (Table 7), as the sequences of the affinity constants K_L_ and of the saturation concentrations *C*_sat_ were identical. According to the *K*_D_ values, TAR was most efficiently absorbed at low initial concentration and GLA at high concentration. The slopes of the Freundlich isotherm parts of GLY and GLU, both > 1.0, indicate a promotion of the adsorption process by already sorbed molecules or complexes.

**Table 8:** Experimental partition coefficients *K*_d_ at initial (P)HCA concentrations of 1.0 and 100 mmol L^−1^ and parameters of the composite Langmuir–Freundlich isotherm for (P)HCA distribution in the gibbsite/ACPW system (pH 13.3)

(P)HCA	*K*_d1,100_^a^ L kg^−1^		*X*^2^/DoF	*r* ^2^	*c*_sat_^b^ ± *S*_D_ mmol kg^−1^	*K*_L_^b^ ± *S*_D_ L mmol^−1^	*K*_F_^b^ ± *S*_D_ mmol^1−*n*^ L^*n*^ kg^−1^	*n*^b^ ± S_D_−
GLY	8.68	0.205	0.53	0.960	8.6 ± 1.6	1.35 ± 0.6	0.01 ± 0.02	1.57 ± 0.36
THR	18.6	n.a.	3.11	0.926	8.1 ± 1.3	4.0 ± 1.6	n.a.	n.a.
TAR	60.5	1.10	1.49	0.959	10.5 ± 4.4	8.77 ± 4.80	5.65 ± 2.02	0.61 ± 0.07
GLU	41.8	1.01	2.66	0.939	20.0 ± 3.2	3.15 ± 1.27	0.45 ± 0.24	1.13 ± 0.10
GLA	30.2	1.75	2.31	0.961	80.7 ± 11.4	0.22 ± 0.06	6.02 ± 1.70	0.52 ± 0.05

*S*_D_, standard deviation; n.a., not applicable

^a^Distribution coefficient *K*_d_ at initial (P)HCA concentrations of 1.0 and 100 mmol L^−1^

^b^Composite Langmuir–Freundlich isotherm parameter (Eq. 2)

## Correlation of sorbate properties and sorption behaviour

For a quantitative discussion, (P)HCA properties were considered, which may be important for the sorption process (Table 9). The concentrations of sorbate molecules forming a complete monolayer at the surface, *C*_surface_, were calculated with respect to their possible surface orientations (parallel or perpendicular). Correspondingly, the maximal (parallel orientation) or the minimal (P)HCA projection area was computed. The constitution of the molecules, i.e. number of functional groups, defines their complex formation ability. An increasing molecule length, reflected by the molecular volume, raises molecular flexibility and the ability to form multidentate and binuclear (bridging) surface complexes. The mean distance between the gibbsite surface sites, assuming five to seven sites per nm^2^, was about 0.5 nm. For comparison, the mean length of a C–C single bond is 0.154 nm and of the C–O bond is 0.143 nm. Thus, vicinal OH or COOH ligand groups are not suited to form bridging surface complexes. Taking the bonding angles into account, the surface coordinating oxygen atoms of a ligand must be separated by a C4 carbon chain at least to be able to form a binuclear complex.

**Table 9:** Specific molecular properties of (P)HCA used for rank correlation

(P)HCA	(P)HCA monolayer concentration		Minimal projection area of PHCA	Maximal projection area of (P)HCA	Dissociation constant of OH next to carboxyl-group	Molecular volume	Molecular mass	No. of negative charges at pH 13.3
	*C*_surface, max_^a^	*C*_surface, min_^a^	PA_min_	PA_max_	pK_OH1_^b^	*V*_vdw_^b^	*M*	*z* ^c^
	mmol kg^−1^		Å^2^		–	Å^3^	g mol^−1^	–
GLY	108.0	70.6	16.9	25.9	14.8	64.5	76.0	1.032
THR	73.5	46.3	24.9	39.5	13.0	115.8	136.1	1.734
TAR	74.3	44.0	24.6	41.5^d^	13.0	117.9	150.1	2.718
GLU	57.2	35.5	31.9	51.5^d^	13.4	167.3	196.2	2.428
GLA	56.2	33.7	32.5	54.2	12.6	169.4	210.1	3.422

^a^Calculation based on maximal/minimal projection area, respectively

^b^From [33]

^c^Calculation based on dissociation constants of functional groups

^d^Data from [23]

The electronic properties depend on the number of functional groups and on their dissociation status. For a given pH of 13.3, we calculated the number of negative charges in the molecules *z* with the help of the R–OH dissociation constants estimated by molecular modelling [33]. These adsorbate properties were correlated with the composite Langmuir–Freundlich isotherm parameters (Table 8) using both Pearson and Spearman rank correlation analyses. The complete correlation matrix is depicted in Table 10.

**Table 10:** Pearson and Spearman rank correlation coefficients of (P)HCA molecular properties and adsorption isotherm parameters

	Pearson correlation coefficient										
	*C*_surf, max_	*C*_surf, min_	PA_min_	PA_max_	pK_OH1_	*V*_vdw_	*M*	*z*	*K*_d1_	*c*_sat_	*K*_L_
Spearman correlation coefficient											
*C*_surf, max_	1.00	*1.00*	***−*** ***0.98***	***−*** ***0.98***	0.87	***−*** ***0.98***	***−*** ***0.98***	− 0.84	0.75	− 0.56	− 0.02
*C*_surf, min_	**0.90**	1.00	***−*** ***0.96***	***−*** ***0.98***	**0.89**	***−*** ***0.96***	***−*** ***0.97***	− 0.87	0.80	− 0.55	− 0.09
PA_min_	***−*** ***1.00***	**−** **0.90**	1.00	*0.99*	− 0.78	*1.00*	*0.99*	0.84	− 0.67	0.65	− 0.15
PA_max_	**−** **0.90**	***−*** ***1.00***	**0.90**	1.00	− 0.81	*1.00*	*1.00*	**0.89**	− 0.72	0.67	− 0.09
pK_OH1_	0.67	0.67	− 0.67	− 0.67	1.00	− 0.76	− 0.80	− 0.84	0.72	− 0.52	− 0.23
*V*_vdw_	**−** **0.90**	***−*** ***1.00***	**0.90**	***1.00***	− 0.67	1.00	*0.99*	0.84	− 0.69	0.65	− 0.13
*M*	**−** **0.90**	***−*** ***1.00***	**0.90**	***1.00***	− 0.67	***1.00***	1.00	**0.90**	− 0.73	0.69	− 0.09
*z*	− 0.70	**−** **0.90**	0.70	**0.90**	− 0.82	**0.90**	**0.90**	1.00	− 0.78	0.75	0.07
*K*_d1_	0.30	0.60	− 0.30	− 0.60	0.36	− 0.60	− 0.60	− 0.70	1.00	− 0.19	− 0.60
*c*_sat_	− 0.70	**−** **0.90**	0.70	**0.90**	− 0.41	**0.90**	**0.90**	0.80	− 0.50	1.00	− 0.56
*K*_L_	0.40	0.30	− 0.40	− 0.30	0.05	− 0.30	− 0.30	− 0.10	− 0.50	− 0.50	1.00

Coefficients emphases indicate significances (italic: 0.001, bold italic: 0.01, bold: 0.05). Parameter as in Tables 8 and 9

The correlation matrix reveals some correlations which are more or less self-evident, e.g. strong positive correlations between molecular mass or molecular volume and the projection areas of the molecules for the two considered orientations at the gibbsite surface. Obviously, the surface concentrations for monolayer coverage were negatively correlated with molecular mass or volume. Interestingly, no significant correlations of the dissociation constant of the most acidic alcohol groups (pk_OH1_), of the linear distribution coefficient (low (P)HCA concentration: *K*_d1_), and the sorption affinity constant (K_L_, composite Langmuir–Freundlich Isotherm, Table 8) with any of the other parameters were ascertained. Most important, the saturation concentration of the Langmuir part of the isotherm (first plateau), *C*_sat_, was positively affected by the size of the sorbats. *C*_sat_ correlated significantly with molecular mass and volume, and with the maximal projection area of the sorbate (Spearman rank correlation coefficients: *r*_s_: 0.90, *p*: 0.05). Moreover, correlations of the Freundlich isotherm parameters with adsorbate charge z were found, albeit with lesser significance, since only four isotherms could be considered.

*K*_F_ correlated positively, whereas the isotherm slope *n* correlated negatively with *z*.

## Discussion

### Adsorption kinetics of d-gluconic acid

More than 90% of the ultimately adsorbed GLU amount was transferred to the gibbsite surface within the first 24 h. Thus, the adaptation of the adsorption experiments to that time span was adequate. Furthermore, no indications for non-ideal sorption modes were found. The equilibrium concentration of the fastly sorbed GLU fraction (2.47 ± 0.27 mmol kg^−1^) matched the concentration of “high affinity sorptions sites” (*C*_sat1_) closely available at that pH (Fig. 2). To the best of our knowledge, no further data on adsorption kinetics of organic acids onto gibbsite have been published.

### Isotherms of d-gluconic acid adsorption at various pH values

The GLU phase distribution data, determined at four pH values, were best fitted by the two-site Langmuir model (Table 3) which is suited to reproduce the S shape of the isotherms. For the same reasons, the two-site Langmuir isotherm was applied in several other investigations concerning anion adsorption, i.e. citrate, fluoride, and antimonate, onto gibbsite and related minerals [34–36]. The assumption that two sorption sites exist, differing in the adsorbate affinity, coincides with the discrimination of the gibbsite surface hydroxyl groups into the more reactive, singly coordinated aluminol groups at the edge faces and into the less reactive, doubly coordinated groups at the basal faces. [14, 15]. Approximately, 5% of the total number of aluminol groups should belong to the reactive type, corresponding to a concentration of 6 mmol kg^−1^ for the aluminium hydroxide used in this study. The bonding capacities of the first type of sites, *C*_sat1_, calculated for pH 4 (6.4 ± 1.5 mmol kg^−1^) and for pH 7 (6.0 ± 1.8 mmol kg^−1^), respectively, match this value very well. At alkaline pH, the adsorbate occupied only about one-third of the reactive sites, suggesting a decreasing availability of these sites with increasing pH. Since the reactive sites are expected to account for the gibbsite surface charge over a wide pH range, it is likely that the decrease of the bonding capacity was caused by electrostatic repulsion between the increasing number of negatively charged sites and the gluconate anions. The low affinity must have been involved in the gluconate adsorption also. This can be inferred from the second Langmuir surface concentration constant *C*_sat2_, which is significantly higher than *C*_sat1_ at every selected pH value. At pH 4 (33 mmol kg^−1^) and pH 7 (19 mmol kg^−1^), the *C*_sat2_ values neither exceeded the GLU monolayer concentration (between 36 and 57 mmol kg^−1^, depending on the GLU surface orientation) nor the total numbers of gibbsite surface sites (120 mmol kg^−1^). These boundaries were surpassed at alkaline pH. The maximal GLU surface concentration (123 mmol kg^−1^) exceeded the calculated GLU monolayer concentration and isotherm fitting did not approximate a *C*_sat2_ value of nearly 120 mmol kg^−1^. This might indicate a change of the sorption mechanism at alkaline pH. As both GLU and the gibbsite surface were negatively charged at that pH, an adsorption mechanism must have occurred overcoming potential electrostatic repulsion between the reaction partners.

### Sorption mechanisms

At pH 4, GLU sorption by electrostatic attraction (outer-sphere complexation) was assumed to prevail, accompanied by H bonding [17]. According to species distribution calculation, 78% of GLU were dissociated, i.e. present as gluconate, and about 30% of the surface sites were positively charged (≡S–OH_2_^+^). Due to the concomitant formation of gluconolactones, the proportion of charged GLU molecules might have been significantly smaller. At pH 7, the carboxylic group of GLU was almost completely dissociated (negatively charged), but the fraction of positively charged surface sites was diminished to about 1% (corresponding to 1.2 mmol kg^−1^) of the total sites. Consequently, the importance of electrostatic sorption was lower at pH 7 than at pH 4. However, at pH 7, gluconate ions might substitute uncharged surface sites via direct ligand exchange (inner-sphere surface complexation) or adsorb by polar interactions, e.g. hydrogen bonding. Due to the predominance of negative surface charges electrostatic attraction between GLU and the surface became less significant at alkaline pH. The monolayer adsorption capacity in the alkaline pH range might have been exceeded due to various processes. Firstly, surface erosion processes, initiated by hydroxide ions and GLU, might have led to an increase of the fractal surface dimension of the initially well-ordered synthetic mineral, thus increasing the number of surface sites. This hypothesis might be proven by various advanced microscopic techniques, including confocal laser scanning microscopy, electron microscopy, and atomic force microscopy. An increase of the surface porosity might be detectable by N_2_ adsorption. Secondly, coordinating Al^3+^ ions might have (partially) compensated the negative charge of solved gluconate ions allowing for adsorption of the formed species. Thirdly, surface precipitation of polynuclear Al complexes, formed during gibbsite hydrolysis, might have enlarged the sorption capacity additionally [37].

### Surface complexation modelling

The interpretation of the types of complexes formed by organic ligands on the surfaces of alumina hydroxides and related minerals is very inconsistent. Some modelling approaches used inner-sphere complexation exclusively to describe the adsorption of various aliphatic and aromatic (hydroxy) acids onto different Al(hydr)oxides in the pH range between 2.5 and 10.8 [15, 34, 38, 39]. Other studies applied combinations of inner- and outer-sphere complexation to model ligand adsorption at acidic and neutral pH [16, 40]. Also, H bonding was considered to contribute to the surface binding of polycarboxylates onto metal oxides [41, 42]. Reflecting these different modelling approaches, our modelling attempt fits well into this spectrum, especially with regard to adsorption at acidic and neutral pH. Deviating from former modelling results, we selected inner-sphere complexation to describe adsorption in the slightly alkaline pH range, leading to an overestimation of the data. No reference data are available for surface complexation modelling at high alkaline pH. With respect to the complex formation properties of GLU in the solution phase [9], we took the formation of various Al(III)–GLU– and Al(III) GLU(OH) complexes into account as well as the formation of ternary surface complexes. With this procedure, it was possible to integrate the unique complexation behaviour of (P)HCAs at high alkaline pH into a surface complexation model.

### Correlation between (P)HCA molecular properties and adsorption behaviour

The high pH value of the ACPW medium exerts several effects on the reaction partners. Two or even three hydroxyl groups of the larger sugar acids get more or less dissociated, generating the prerequisites for tridentate Al(III) coordination in solution [9, 23]. With increasing pH, the carboxylate group is replaced in the coordination sphere by an alcoholate group. The free carboxylate group is available both for surface bonding via a ligand exchange reaction or for interaction with a second Al(III) ion. In the latter case, ternary surface complex formation by a bridging Al(III) ion would be possible. Polyhydroxy dicarboxylic acids are assumed to form polynuclear complexes with Al ions bridging between the (P)HCA units. It is also likely that these complexes were adsorbed too. These additional reaction modes might contribute to the differences in the isotherm shapes between the mono- and dicarboxylic acids (Fig. 4). In contrast to the monocarboxylic acids, the sorption functions of the dicarboxylic acids could be fitted by the two-site Langmuir approach, resulting in high determination coefficients.

Due to the relatively high gibbsite solubility at pH 13.3 [theoretical Al(III) equilibrium concentration: approximately 0.1 mol L^−1^], complex formation with these ions is especially favoured at low and moderate (P)HCA initial concentrations. The analysis of Al(III) ions in the ACPW medium at established (P)HCA distribution equilibrium showed a negative correlation with (P)HCA concentrations up to a ligand concentration of roughly 20 mmol L^−1^ (data not shown). This indicates a concerted elimination of both solutes from the solution. Furthermore, the comparison of GLU adsorption at pH 12 (Fig. 3) and pH 13.3 (Fig. 4) elucidates a higher sorption in the ACPW medium under conditions, where the Al(III) concentrations surpassed that of GLU (≤ 20 mmol L^−1^ GLU). The isotherms nearly coincide at higher GLU concentrations.

Ca^2+^ ions (concentration in ACPW: 2 mmol L^−1^) might also be able to interfere with (P)HCA adsorption and complexation reactions, at least at low ligand concentrations. The formation of simple CA–(P)HCA complexes can be neglected because of the considerable excess of Al(III) ions which form (P)HCA complexes of superior stability [43]. Nevertheless, the occurrence of mixed binuclear complexes like [CaAlH_−5_GLU]^−^, as it is reported for analogous Ca^2+^–Fe^3+^ complexes, has to be taken into account [44].

The high alkaline pH led to a levelling of the reactivity differences of singly and doubly coordinated aluminol groups. Thus, differing from GLU adsorption at acidic or neutral pH (Fig. 2), no (first) surface saturation (*C*_sat(1)_) close to 6 mmol kg^−1^, corresponding to the surface concentration of the singly coordinated aluminols, was found, independently of the applied isotherm type. A high reactivity of the gibbsite basal planes towards polyols and sugar acids was previously assumed to explain inhibited crystal growth and altered crystal morphologies after addition of these ligands to concentrated sodium aluminate liquors (“Bayer liquor”) [22]. According to the correlation matrix (Table 10), the *C*_sat_-value from the composite Langmuir–Freundlich isotherm correlated positively with the (P)HCA size. Clearly, the suitability for tridentate Al chelation and additional metal and/or surface interactions increases with an increasing number of functional groups and increasing steric arrangement options. But this alone would not explain the fourfold higher *C*_sat_ of GLA compared with GLU. Together with the above-mentioned ability to form polynuclear complexes, further binding options of dicarboxylates have to be considered, given that the steric constraints are fulfilled: firstly, at low dicarboxylate:sorption site ratio, bidentate surface binding via both carboxylate groups; secondly, at high ligand:site ratio, multilayer formation by coordination of solved Al–(hydroxo)-dicarboxylate chelates to already adsorbed ligands and Al chelates via free carboxylate groups or by Al(III) bridging. Multilayer formation is a potential explanation for the high *C*_sat_ value of GLA, which exceeded the maximum ligand concentration that fit into one surface layer. This hypothesis, together with surface precipitation of Al–(P)HCA chelates, is also useful to interpret GLU and TAR surface concentrations at PHCA equilibrium concentrations ≥ 0.1 mol L^−1^.

## Conclusions

The investigation of the adsorption of (poly) hydroxy carboxylic acids on gibbsite revealed strong dependencies on the pH value and on molecular features of the acids. The modelling of the S-shaped GLU sorption edge (maximum around pH 4, minimum around pH 10) with a surface complexation approach resulted in a fair agreement in the acidic and neutral pH range, but led to considerable differences in the moderately alkaline pH range, indicating the formation and phase distribution of adsorbate species which were not considered by the model.

The analysis of correlation between parameters of several adsorption isotherms and specific molecular adsorbate properties, conducted for adsorption in artificial cement pore water at pH 13.3, showed a high importance of the size and thereby of the number of functional groups.

Three adsorption mechanisms were indicated, which effected the (P)HCA adsorption over the whole pH range, albeit with different importance: electrostatic interaction as primary mechanism at low pH, hydrophilic interactions, i.e. hydrogen bonding, dominating at intermediate pH, and inner-sphere complex formation, including ternary complex formation, at high pH. Sorption in two stages pointed to the involvement of different sorption sites and different sorption mechanisms. At low pH, sorption behaviour of GLU was comparable to that of polycarboxylic acids, but the suggested mechanisms were different. In the alkaline pH range, the sorption behaviour of GLU differed from polycarboxylic acids but was similar to that of polyols, suggesting a significant contribution of partially deprotonated hydroxyl groups to the sorption process. Thus, we conclude that the sorption behaviour of GLU and of structurally related (P)HCA is best described by a combination of the adsorption properties of (poly)carboxylic acids at acidic pH and polyols at alkaline pH.

## Abbreviations

PHCA: polyhydroxy carboxylic acids
ACPW: artificial cement pore water
pH_IEP_: pH value at isoelectric point
GLY: glycolic acid
THR: d-threonic acid
TAR: l-tartaric acid
GLU: d-gluconic acid
GLA: d-glucaric acid
DRIFT: diffuse reflectance infrared Fourier transform (spectroscopy)

## References

[1] Moghimi A, Tate ME, Oades JM (1978). Characterization of rhizosphere products, especially 2-ketogluconic acid. Soil Biol Biochem.

[2] Bar-Yosef B, Rogers RD, Wolfram JH, Richman E (1999). *Pseudomonas cepacia*-mediated rock phosphate solubilization in kaolinite and montmorillonite suspensions. Soil Sci Soc Am J.

[3] Glaus MA, van Loon LR, Achatz S, Chodura A, Fischer K (1999). Degradation of cellulosic materials under the alkaline conditions of a cementitious repository for low and intermediate level radioactive waste. Part I: identification of degradation products. Anal Chim Acta.

[4] Askarieh MM, Chambers AV, Daniel FBD, Fitzgerald PL, Holtom GJ, Pilkington NJ, Rees JH (2000). The chemical and microbial degradation of cellulose in the near field of a repository for radioactive wastes. Waste Manage.

[5] Knill CJ, Kennedy JF (2003). Degradation of cellulose under alkaline conditions. Carbohydr Polym.

[6] Rout SP, Charles CJ, Doulgeris C, McCarthy AJ, Loughnane JP, Laws AP, Humphreys PN (2015). Anoxic biodegradation of isosaccharinic acids at alkaline pH by natural microbial communities. PLoS ONE.

[7] Sawyer TD (1964). Metal–gluconate-complexes. Chem Rev.

[8] Velasco JG, Ortega J, Sancho J (1976). On the composition and stability of some D-(+) saccharic acid complexes. J Inorg Nucl Chem.

[9] Motekaitis RJ, Martell AE (1984). Complexes of aluminum(III) with hydroxy carboxylic acids. Inorg Chem.

[10] Best WM, Harrowfield JM, Shand TM, Stick RV (1994). Aluminum(III) coordination to hydroxy carboxylates: the influence of hydroxy substituents enabling tridentate binding. Aust J Chem.

[11] Stockdate A, Bryan ND (2013). The influence of natural organic matter on radionuclide mobility under conditions relevant to cementitious disposal of radioactive wastes: a review of direct evidence. Earth Sci Rev.

[12] Dagnelie RVH, Descostes M, Pointeau I, Klein J, Grenut B, Radwan J, Lebeau D, Georgin D, Giffaut E (2014). Sorption and diffusion of organic acids through clayrock: comparison with inorganic anions. J Hydrol.

[13] Digne M, Sautet P, Raybaud P, Toulhoat H, Artacho E (2002). Structure and stability of aluminum hydroxides: a theoretical study. J Phys Chem.

[14] Hiemstra T, de Wit JC, van Riemsdijk WH (1989). Multisite proton adsorption modeling at the solid/solution interface of (hydr)oxides: a new approach. II. Application to various important (hydr)oxides. J Colloid Interface Sci.

[15] Essington ME, Anderson RM (2008). Competitive adsorption of 2-ketogluconate and inorganic ligands onto gibbsite and kaolinite. Soil Sci Soc Am J.

[16] Nordin J, Persson P, Laiti E, Sjöberg S (1997). Adsorption of o-phthalate at the water-boehmite (γ-AlOOH) interface: evidence for two coordination modes. Langmuir.

[17] Nordin J, Persson P, Nordin A, Sjöberg S (1998). Inner-sphere and outer-sphere complexation of a polycarboxylic acid at the water-boehmite (γ-AlOOH) interface: a combined potentiometric and IR spectroscopic study. Langmuir.

[18] Rosenqvist J, Axe K, Sjöberg S, Persson P (2003). Adsorption of dicarboxylates on nano-sized gibbsite particles: effects of ligand structure on bonding mechanisms. Colloids Surf A.

[19] Alliot C, Bion L, Mercier F, Toulhoat P (2005). Sorption of aqueous carbonic, acetic, and oxalic acids onto α-alumina. J Colloid Interface Sci.

[20] Pommerenk P, Schafran GC (2005). Adsorption of inorganic and organic ligands onto hydrous aluminum oxide: evaluation of surface charge and the impacts of particle and NOM removal during water treatment. Environ Sci Technol.

[21] Singh K, Mohan S (2004). Adsorption behaviour of selected monosaccharides onto alumina surface. J Colloid Interface Sci.

[22] van Bronswijk W, Watling HR, Yu Z (1999). A study of the adsorption of acyclic polyols on hydrated alumina. Colloids Surf A.

[23] Coyne JF, Wainwright MS, Cant NW, Grocott SC (1994) Adsorption of hydroxy organic compounds on alumina trihydrate. In: Proceedings of the 123^rd^ TMS annual meeting on light metals - San Francisco, CA, USA. Edited by the Minerals, Metals & Materials Society (TMS), pp 39–45

[24] Baeyens B, Bradbury MH (1997). A mechanistic description of Ni and Zn sorption on Na-montmorillonite. Part I: titration and sorption measurements. J Contam Hydrol.

[25] Berner U (1990) A thermodynamic description of the evolution of pore water chemistry and uranium speciation during the degradation of cement. PSI Report 62, Paul Scherrer Institute, Würenlingen and Villigen, Switzerland

[26] Nagy NM, Kónya J (2010). Interfacial chemistry of rocks and soils.

[27] Deshpande PA, Polisetti S, Madras G (2011). Rapid synthesis of ultrahigh adsorption capacity zirconia by a solution combustion technique. Langmuir.

[28] Xing B, Pignatello JJ (1997). Dual-mode sorption of low polarity compounds in glassy poly(vinyl chloride) and soil organic matter. Environ Sci Technol.

[29] Schneckenburger T, Schaumann GE, Woche SK, Thiele-Bruhn S (2012). Short-term evolution of hydration effects on soil organic matter properties and resulting implications for sorption of naphthalene-2-ol. J Soils Sediments.

[30] Davis JA, Kent DB (1990) Surface complexation modeling in aqueous geochemistry. In: Hochella MF, White AF (eds) Mineral–water interface geochemistry. Reviews in mineralogy 5, pp 177–260

[31] Karamalidis AK, Dzombak DA (2010). Surface complexation modelling.

[32] Stumm W, Morgan JJ (1996). Aquatic chemistry: an introduction emphasizing chemical equilibria in natural waters.

[33] Chemicalize – online platform for chemical calculations, search, and text processing. https://chemicalize.com/. Accessed 12 Feb 2015

[34] Hidber PC, Graule TJ, Gauckler LJ (1996). Citric acid—a dispersant for aqueous alumina suspensions. J Am Ceram Soc.

[35] Chai LY, Wang YY, Zhao N, Yang WC, You XY (2013). Sulfate-doped Fe_3_O_4_/Al_2_O_3_ nanoparticles as a novel adsorbent for fluoride removal from drinking water. Water Res.

[36] Essington M (2013) Antimony(V) adsorption by variable-charge minerals. Report SERDP Project ER-1741

[37] Adekola F, Fédoroff M, Geckeis H, Kupcik T, Lefèvre G, Lützenkirchen J, Plaschke M, Preocanin T, Rabung T, Schild D (2011). Characterization of acid-base properties of two gibbsite samples in the context of literature results. J Colloid Interface Sci.

[38] Benoit P, Hering JG, Stumm W (1993). Comparative study of the adsorption of organic ligands on aluminum oxide by titration calorimetry. Appl Geochem.

[39] Xiao-hong G, Guang-hao C, Chii S (2007). ATR-FTIR and XPS study on the structure of complexes formed upon the adsorption of simple organic acids on aluminum hydroxide. J Environ Sci.

[40] Persson P, Nordin J, Rosenqvist J, Lövgren L, Öhman L-O, Sjöberg S (1998). Comparison of the adsorption of phthalate on boehmite (γ-AlOOH), aged Al_2_O_3_, and goethite (α-FeOOH). J Coll Interface Sci.

[41] Lindegren M, Persson P (2009). Competitive adsorption between phosphate and carboxylic acids: quantitative effects and molecular mechanisms. Europ J Soil Sci.

[42] Johnson SE, Sjoberg S, Persson P (2004). Surface complexation of mellitic acid to goethite: an attenuated total reflectance Fourier transform infrared study. Langmuir.

[43] Smith RM, Martell AE, Motekaitis RJ (2001). NIST critically selected stability constants of metal complexes database. NIST Standard Reference Database 46, version 6.0..

[44] Bechtold T, Burtscher E, Turcanu A (2002) Ca^2+^-Fe^3+^-d-gluconate-complexes in alkaline solution. Complex stabilities and electrochemical properties. J Chem Soc Dalton Trans 2683–2688

